# *Map2k7* Haploinsufficiency Induces Brain Imaging Endophenotypes and Behavioral Phenotypes Relevant to Schizophrenia

**DOI:** 10.1093/schbul/sbz044

**Published:** 2019-06-20

**Authors:** Rebecca L Openshaw, David M Thomson, Rhiannon Thompson, Josef M Penninger, Judith A Pratt, Brian J Morris, Neil Dawson

**Affiliations:** 1 Institute of Neuroscience and Psychology, College of Medical, Veterinary, and Life Sciences, University of Glasgow, Glasgow, UK; 2 Strathclyde Institute of Pharmacy and Biomedical Science, University of Strathclyde, Glasgow, UK; 3 Institute for Molecular Biotechnology of Austrian Academy of Sciences (IMBA), Vienna, Austria; 4 Division of Biomedical and Life Sciences, Faculty of Health and Medicine, Lancaster University, Lancaster, UK

**Keywords:** network science, preclinical models, functional brain imaging

## Abstract

c-Jun N-terminal kinase (JNK) signaling contributes to functional plasticity in the brain and cognition. Accumulating evidence implicates a role for MAP kinase kinase 7 (MAP2K7), a JNK activator encoded by the *Map2k7* gene, and other JNK pathway components in schizophrenia (ScZ). Mice haploinsufficient for *Map2k7 (Map2k7*^*+/−*^ mice) display ScZ-relevant cognitive deficits, although the mechanisms are unclear. Here we show that *Map2k7*^*+/−*^ mice display translationally relevant alterations in brain function, including hippocampal and mesolimbic system hypermetabolism with a contrasting prefrontal cortex (PFC) hypometabolism, reminiscent of patients with ScZ. In addition *Map2k7*^*+/−*^ mice show alterations in functional brain network connectivity paralleling those reported in early ScZ, including PFC and hippocampal hyperconnectivity and compromised mesolimbic system functional connectivity. We also show that although the cerebral metabolic response to ketamine is preserved, the response to dextroamphetamine (d*-*amphetamine) is significantly attenuated in *Map2k7*^*+/−*^ mice, supporting monoamine neurotransmitter system dysfunction but not glutamate/NMDA receptor (NMDA-R) dysfunction as a consequence of *Map2k7* haploinsufficiency. These effects are mirrored behaviorally with an attenuated impact of d*-*amphetamine on sensorimotor gating and locomotion, whereas similar deficits produced by ketamine are preserved, in *Map2k7*^*+/−*^ mice. In addition, *Map2k7*^*+/−*^ mice show a basal hyperactivity and sensorimotor gating deficit. Overall, these data suggest that *Map2k7* modifies brain and monoamine neurotransmitter system function in a manner relevant to the positive and cognitive symptoms of ScZ.

## Introduction

c-Jun N-terminal kinase (JNK) signaling plays a key role in synaptic plasticity and neuronal communication.^1^ In humans, mutations in JNK genes lead to intellectual disability.^2^ We have identified a common variant in the *MAP2K7* gene (encoding MAP kinase kinase 7 (MAP2K7), which activates JNKs) that shows a strong association with schizophrenia (ScZ).^3^ Genes in this signaling pathway are independently associated with ScZ (supplementary figure S1).^4,5^ Hence, strong evidence implicates the pathways modulated by MAP2K7 in ScZ. Furthermore, *MAP2K7* transcript levels are reduced in the prefrontal cortex (PFC) of ScZ patients^3^ and *Map2k7* haploinsufficient mice (*Map2k7*^+/*−*^ mice) show deficits in PFC-dependent tasks.^3,6^ Despite these observations we have a poor understanding of how *MAP2K7* mutations increase the risk of developing ScZ.

Identifying neurobiological biomarkers with translational parallels in patients and rodent models is a major objective in psychiatric drug discovery. For ScZ the greatest progress has arguably been made for cognitive symptoms.^7–9^ Developing translatable tests for positive and negative symptoms is more challenging. Prepulse inhibition (PPI) is widely promoted as a cross-species measure recruiting similar neural circuitry and reflecting neural domains involved in positive and cognitive symptoms.^10,11^ Impaired PPI is robustly observed in ScZ and correlates with positive symptomatology.^12–15^ Other rodent behaviors deemed relevant to positive symptoms include hyperlocomotion, potentially indicative of dopaminergic hyperfunction.^11^ Although the translational relevance of hyperlocomotion is debated, it is influenced by ScZ risk genes^16,17^ and is present in patients.^18^

ScZ patients exhibit a characteristic pattern of brain imaging (functional and structural) and neurochemical abnormalities. In chronic ScZ functional abnormalities include reduced PFC activity (hypofrontality).^19,20^ However, during acute psychosis patients show a different profile with elevated ventral striatum (nucleus accumbens) and hippocampus/temporal cortex activity.^21–23^ Hippocampal/temporal cortex hyperactivity is also seen in chronic ScZ and in those at high risk of psychosis.^24^ Many studies have characterized alterations in functional brain network and regional connectivity in ScZ, with complex and contradictory findings. In general these studies support enhanced functional connectivity, potentially limited to specific neural systems, during early stages of the disorder^25–28^ with a contrasting reduction in connectivity in chronic ScZ.^29–31^ Dysconnectivity in ScZ may be both disease time-course and neural system dependent. For example, with regard to neural systems, increased accumbens-PFC,^32,33^ reduced ventral tegmental area (VTA)–thalamus,^34^ reduced VTA–insular cortex,^35^ and increased hippocampal–PFC connectivity^36^ have been reported. Equivalent phenotypes are rarely studied in rodent models. However, we have shown hypofrontality in rats following subchronic NMDA-R antagonist administration^37,38^ and in mice with truncated *Disc1*.^39^ These models also show brain network connectivity alterations relevant to ScZ.^38–40^ These phenotypes provide mechanistic insight and offer translational biomarkers against which the efficacy of novel therapeutics can be tested.^41^

Evidence supports both glutamatergic and monoamine (dopamine, serotonin, and noradrenaline) system dysfunction in ScZ,^42–45^ with glutamatergic/NMDA-R hypofunction and monoamine hyperfunction, particularly for dopamine, supported. These disturbances have also been linked to the dysfunction of ScZ risk genes, including *Disc1.*^39,46^ Characterizing cerebral metabolic responses to drugs that challenge these systems provides insight into their in vivo function and the mechanisms by which ScZ risk genes mediate their effect.

Here we test the hypothesis that *Map2k7*^*+/−*^ mice exhibit alterations in cerebral metabolism and functional brain network connectivity relevant to ScZ. To elucidate the impact of *Map2k7* haploinsufficiency on in vivo NMDA-R/glutamate and monoamine system function we characterize the cerebral metabolic response to ketamine and dextroamphetamine (d*-*amphetamine), drugs that induce ScZ-like symptoms in humans. In light of the results obtained, we also characterize the impact of d*-*amphetamine and ketamine on PPI and locomotor activity (LMA) in *Map2k7*^*+/−*^ mice.

## Methods

### Animals

Mice heterozygous for *Map2k7* (*Map2k7*^+/*−*^) were produced as previously described^47^ and backcrossed onto the C57BL/6 background.^6^ Animals were house under standard conditions (21^o^C, 45%–65% humidity) with a 12-hour light/dark cycle (lights on 08:00). Experiments were conducted in accordance with the UK Animals (Scientific Procedures) Act 1986.

### ^14^C-2-Deoxyglucose Functional Brain Imaging

^14^C-2-Deoxyglucose (^14^C-2-DG) brain imaging was completed in *Map2k7*^*+/−*^ and wild-type (WT) littermate mice as previously described.^39,48^ Alterations in local cerebral glucose utilization (LCGU) in brain regions of interest (RoI) were analyzed using ANOVA with sex and genotype as independent variables. Significance was set at *P* < .05.

### Cerebral Metabolic Responses to d*-*amphetamine and Ketamine

*Map2k7* ^+/*−*^ and WT mice were challenged with d*-*amphetamine (5 mg/kg, intraperitoneally (i.p.), WT: male *n* = 5, female *n* = 6; *Map2k7*^+/*−*^: male, *n* = 5; female *n* = 6) or ketamine (25mg/kg, i.p., WT: male *n* = 4, female *n* = 4; *Map2k7*^+/*−*^: male, *n* = 5; female *n* = 5) in physiological saline (2 ml/kg) and underwent the ^14^C-2-DG protocol.^39,49^ In accordance with published protocols^14^C-2DG was injected 1 minute after ketamine and 15 minutes after d*-*amphetamine.^39,49,50^ Control animals received saline (i.p., WT: male *n* = 8, female *n* = 10; *Map2k7*^+/*−*^: male *n* = 5; female *n* = 7) either 1 minute or 15 minutes before ^14^C-2-DG (50% sample for each time). Data were analyzed using ANOVA with sex, genotype and treatment as independent variables. Significance was set at *P* < .05.

### Functional Brain Network Analysis

Brain network properties were determined through analysis of the data from saline-treated animals. Global brain network properties, including mean degree (<*k*>), the clustering coefficient (C_p_) and average path length (L_p_) were determined as previously described.^39,40,48^ Regional importance was defined through centrality analysis (degree [K_i_], betweenness [B_i_], closeness [C_i_], and eigenvector centrality [E_i_]) as previously described.^39,40,48^ The standardized *z*-score centrality measure for each RoI was calculated by comparison to 11 000 calibrated Erdös−Rényi networks. The significance of the *z*-score difference between experimental groups was determined by comparison to 55 000 random permutations of the data. Significance was set a *P* < .05. A composite *z*-score, across all centrality measures, was also determined with a difference >1.96 or < −1.96 considered significant. A detailed overview of the network analysis approaches used is included in the supplementary material.

### Analysis of Regional Functional Connectivity

Regional functional connectivity was analyzed using partial least squares regression (PLSR) as previously described^38,39^ using the PLS package^51^ in R (R Core Team, 2018). “Seed” region connectivity to all other RoI (57) was defined by the variable importance to the projection (VIP) statistic. The VIP, SD, and confidence interval (CI) were estimated by jack-knifing. A significant connection between regions exist if the 95% CI of the VIP statistic >0.8.^52^ Genotype-induced alterations in the VIP statistic were statistically determined by the standardized *z*-score (*z*-score > 1.96 or < −1.96 considered significant).

### Prepulse Inhibition

PPI was measured in SR-LAB chambers (San Diego Instruments) as previously described.^53^ The impact of 5 mg/kg d*-*amphetamine i.p. (WT: male *n* = 11, female *n* = 6; *Map2k7*^*+/−*^: male *n* = 11, female *n* = 12) and 25 mg/kg ketamine i.p. (WT: male *n* = 5, female *n* = 4; *Map2k7*^*+/−*^: male *n* = 3, female *n* = 4) on % PPI was determined, as these doses disrupt PPI in WT mice.^54,55^ Drug and vehicle treatment were counterbalanced across days in each experiment. Thereafter % PPI was calculated [(startle reactivity at 120 dB – startle reactivity with prepulse) / startle reactivity at 120 dB × 100] and analyzed by ANOVA with genotype and sex as between subjects factors, prepulse intensity and treatment as within subjects factors and each mouse nested within genotype and sex. Pairwise comparisons were made using Fisher’s post hoc test.

### Locomotor Activity

LMA was monitored in black opaque Perspex XT arenas (40 × 40 × 40 cm) lit from below by infrared LEDs. The impact of d-amphetamine and ketamine on LMA was determined, with drug and vehicle injection counterbalanced across days in each experiment. Each mouse was placed in the center of the arena and allowed to explore for 15 minutes (habituation). Mice were then treated with 3 mg/kg d-amphetamine i.p. (WT: male *n* = 5, female *n* = 4; *Map2k7*^*+/−*^: male *n* = 3, female *n* = 4), 20 mg/kg ketamine i.p. (WT: male *n* = 5, female *n* = 5; *Map2k7*^*+/−*^: male *n* = 5, female *n* = 5) or saline and placed in the arena for the test period (ketamine, 30 min; d-amphetamine, 60 min). Total distance moved (cm) was measured using EthoVision XT (Noldus Information Technology) and analyzed by ANOVA with genotype, sex and treatment as between subjects factors and each mouse nested within genotype and sex. Pairwise comparisons were made using Fisher’s post hoc test.

## Results

### *Map2k7* Haploinsufficient Mice Show Hippocampal and VTA Hypermetabolism and Orbital Cortex Hypometabolism

**Fig 1.**
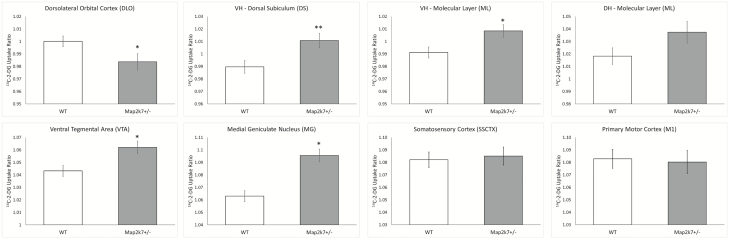
Constitutive LCGU is altered in *Map2k7*^*+/−*^ mice. *Map2k7*^*+/−*^ mice show dorsolateral orbital cortex (DLO) hypometabolism and hippocampal (dorsal subiculum, VH-DS; molecular layer, VH-ML), ventral tegmental area (VTA) and medial geniculate (MG) hypermetabolism. Data shown as mean ± standard error of the mean. *P < .05, **P < .01 genotype effect (ANOVA).

*Map2k7* ^*+/−*^ mice show hippocampal hypermetabolism, with increased LCGU in the dorsal subiculum (VH-DS, *F*_(1,26)_ = 7.78, *P* = .009) and molecular layer (VH-ML, *F*_(1,26)_ = 6.59, *P* = .016) of the ventral hippocampus (figure 1). LCGU was also increased in the VTA (*F*_(1,26)_ = 4.58, *P* = .041) and medial geniculate (MG, *F*_(1,26)_ = 4.25, *P* = .049) of *Map2k7*^*+/−*^ mice. By contrast, LCGU was significantly decreased in the dorsolateral orbital cortex (DLO, *F*_(1,26)_ = 6.06, *P* = .021) of *Map2k7*^*+/−*^ mice. We found no evidence that the impact of *Map2k7* haploinsufficiency on LCGU was influenced by sex. Full data shown in supplementary table S1.

### *Map2k7* Haploinsufficient Mice Show Abnormal Functional Brain Network Connectivity Supporting Hippocampal and Septum/DB Hyperconnectivity and Mesolimbic Hypoconnectivity

**Fig. 2.**
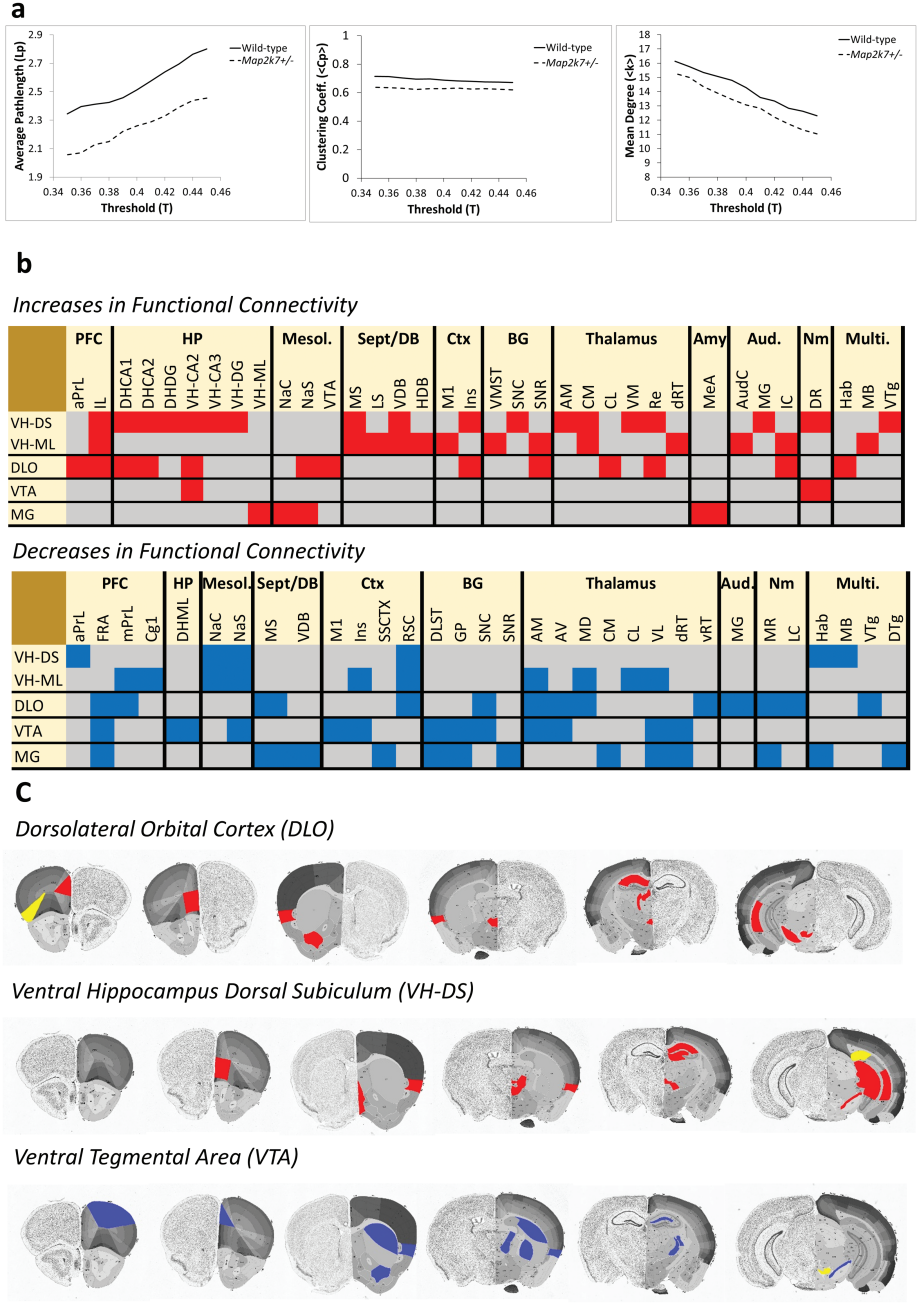
*Map2k7* ^*+/−*^ mice show altered functional brain network connectivity. (**a**) Average path length (L_p_) is decreased (*P* = .012) whereas mean degree (<*k*>) and clustering (C_p_) are not altered in *Map2k7*^*+/−*^ mice. (**b**) Heatmaps showing altered inter-regional connectivity in *Map2k7*^*+/−*^ mice. Red denotes gained (ventral tegmental area [VIP] 95% CI >0.8 in *Mapk7*^*+/−*^, <0.8 in wild-type [WT] and *z*-score difference >1.96) and blue denotes lost (VIP 95% CI >0.8 in WT, <0.8 in *Map2k7*^*+/−*^ and *z*-score difference <−1.96) connectivity in *Map2k7*^*+/−*^ mice. PFC = prefrontal cortex, HP = hippocampus, Mesol. = mesolimbic system, Sept/DB = septal/diagonal band of Broca, Ctx = cortex, BG = basal ganglia, Aud. = auditory system, Nm = neuromodulatory nuclei, Multi. = multimodal. Full connectivity heatmaps are shown in supplementary figure S2. (**c**) Brain images showing the anatomical localization of altered inter-regional connectivity in *Map2k7*^*+/−*^ mice. Blue denotes lost and red denotes gained connectivity. Brain sections modified from the Allen atlas (mouse.brain-map.org/static/atlas).

Globally, functional brain networks in *Map2k7*^+/*−*^ mice had a similar number of connections (<*k*>) and showed a similar level of clustering (C_p_) to those in WT animals. However, average path length (L_p_) is significantly reduced (*P* = .0123) in *Map2k7*^+/*−*^ mice (figure 2), supporting an altered network structure that promotes more efficient information transfer across brain networks in these animals.

Multiple hippocampal subfields were identified as having significantly greater centrality in *Map2k7*^+/*−*^ mice (table 1). Multiple regions of the septum/diagonal band of Broca (septum/DB) and amygdala, along with the dorsal reticular thalamus (dRT) and multiple PFC subfields, were also identified as having increased centrality in *Map2k7*^+/*−*^ mice. These findings support the hyperconnectivity of these neural systems in *Map2k7*^+/*−*^ mice, that contributes to the global hyperconnectivity (decreased L_p,_figure 2) seen in these animals. Significant decreases in centrality in *Map2k7*^+/*−*^ mice were also identified, limited to the mesolimbic system and retrosplenial cortex (RSC, table 1). Full centrality data shown in supplementary table S2.

**Table 1.** Alterations in Regional Centrality in *Map2k7*^*+/−*^ Mice

Brain Region	Degree (K_i_)	Betweenness (B_i_)	Closeness (C_i_)	Eigenvector (E_i_)	Composite *Z*-score
**Increased centrality**					
Prefrontal cortex					
aPrL	2.34	3.14	3.79	2.64	2.98
DLO	1.71	7.21	3.72	0.80	3.36
IL	0.29	2.40	6.79*	3.00*	3.12
Septum/diagonal band of broca					
MS	2.51	3.15	7.05*	5.72*	4.61
LS	2.28	3.08	7.12*	5.69*	4.54
HDB	2.18	1.83	7.30*	3.29*	3.65
Hippocampus					
DH-CA1	1.01	0.68	5.13	6.21*	3.26
DH-CA2	1.29	0.33	5.35	6.08*	3.26
DH-DG	0.62	−1.15	4.43	5.94*	2.46
VH-CA1	2.05	−1.23	3.68	5.85*	2.59
VH-CA2	1.51	0.44	3.86	5.44*	2.81
VH-CA3	1.24	1.14	4.71	6.23*	3.33
VH-DG	0.73	0.92	4.24	5.77*	2.91
Amygdala					
BLA	1.87	1.74	5.61	5.78*	3.75
MeA	0.76	−0.08	3.57	4.91*	2.29
Thalamus					
dRT	1.15	5.12	5.84*	−0.36	2.94
**Decreased centrality**					
Mesolimbic system					
NaC	−3.06	−14.54*	−2.68	0.15	−5.03
NaS	−0.20	−5.31	−2.72	−0.02	−2.06
VTA	−1.60	−0.92	−1.10	−4.26	−1.97
Cortex					
RSC	−1.21	−5.53	0.20	−3.46	−2.50
Piri	−1.85	−14.89*	−1.33	−3.70	−5.44
FRA	−2.72	−3.80	0.07	−2.58	−2.26

*Note*: aPrL, anterior prelimbic cortex; DLO, dorsolateral orbital cortex; IL, infralimbic cortex; MS, medial septum; LS, lateral septum; HDB, horizontal limb of the diagonal band of Broca; DH-CA1, dorsal hippocampus cornu ammonis 1; DH-CA2, dorsal hippocampus cornu ammonis 2; DH-DG, dorsal hippocampus dentate gyrus; VH-CA1; ventral hippocampus cornu ammonis 1; VH-CA2, ventral hippocampus cornu ammonis 2; VH-CA3, ventral hippocampus cornu ammonis 3, VH-DG, ventral hippocampus dentate gyrus; BLA, basolateral amygdala; MeA, medial amygdala; dRT, dorsal reticular thalamus, NaC, nucleus accumbens core; NaS, nucleus accumbens shell; VTA, ventral tegmental area; RSC, retrosplenial cortex; Piri, piriform cortex, FRA, frontal association cortex.

*denotes *P* < .05 significant difference from WT (55 000 random permutations of the real data) within the given centrality measure. A composite standardized *z*-score was also calculated across all centrality measures. A composite *z*-score > 1.96 or < −1.96 was considered to be significant. Positive *z*-scores indicate an increase in regional centrality in *Map2k7*^*+/−*^mice relative to WT controls, and negative *z*-scores indicate a decrease in centrality in *Map2k7*^*+/−*^mice relative to controls. Only regions with a significant composite *z*-score are shown. Full centrality data shown in supplementary table S4.

### PLSR Analysis Further Identifies Altered Prefrontal–Thalamic–Hippocampal, Septum/DB-Hippocampal and Mesolimbic System Connectivity in *Map2K7*^*+/−*^ Mice

PLSR analysis revealed prefrontal–thalamic–hippocampal and septum/DB–hippocampal hyperconnectivity, along with mesolimbic system hypoconnectivity, in *Map2k7*^+/*−*^ mice (figure 2) mirroring the alterations found by centrality analysis (table 1).

When the ventral hippocampus DS and ML were considered as seed regions there was evidence that the connectivity of the septum/DB, which provides direct innervation to the hippocampus,^56^ was abnormally increased in *Map2k7*^+/*−*^ mice (figure 2), consistent with the increased centrality of these regions in these animals. The connectivity of DS and ML to the medial PFC (infralimbic cortex, IL) was also significantly increased in *Map2k7*^+/*−*^ mice. There was also evidence for enhanced connectivity between hippocampal subfields in *Map2k7*^+/*−*^ mice, specifically for the DS, a primary output of the hippocampus.^57^ Other abnormal DS and ML connectivity in *Map2k7*^+/*−*^mice included increased connectivity to the thalamus (anteromedial nucleus [AM], centomedial nucleus [CM], ventromedial nucleus [VM], nucleus reuniens [Re]), auditory system (auditory cortex [AudC], medial geniculate [MG], inferior colliculus [IC]) and the serotonergic dorsal raphé (DR). In contrast to these broad increases in connectivity, selective losses in DS and ML connectivity were also found, including lost connectivity to the mesolimbic system (nucleus accumbens core [NaC], nucleus accumbens shell [NaS]) and RSC, mirroring the decreased centrality of these regions (table 1).

When the DLO was considered as the seed region abnormal functional connectivity between this PFC subfield and the hippocampus (DH-CA1, DH-CA2, VH-CA2) was found in *Map2k7*^*+/−*^ mice. In addition, the DLO showed abnormal functional connectivity to the mesolimbic system (VTA and NaS) in *Map2k7*^*+/−*^ mice. By contrast, connectivity of the DLO to its projecting thalamic nuclei (AM, anteroventral nucleus [AV], mediodorsal nucleus [MD]) was lost in *Map2k7*^*+/−*^ mice.

When the VTA was considered as the seed, lost connectivity to other mesolimbic regions (NaS), the basal ganglia (dorsolateral striatum [DLST], globus pallidus [GP], substantia nigra pars compacta [SNC]) and thalamic nuclei (AM, AV, ventrolateral nucleus [VL], dRT) was found in *Map2k7*^*+/−*^ mice. Significant increases in VTA connectivity in *Map2k7*^*+/−*^ mice were limited. However, new abnormal connectivity between the DR and VTA was found, suggesting that serotonergic regulation of the VTA may be altered in *Map2k7*^*+/−*^ mice (figure 2).

MG functional connectivity was also altered in *Map2k7*^*+/−*^ mice, including increased connectivity to the mesolimbic system (NaC and NaS) and lost connectivity to the basal ganglia (DLST, GP, SNR) and thalamic nuclei (dRT, CM and VL).

Full PLSR data are included in supplementary tables S3–S7.

### The Cerebral Metabolic Response to Ketamine Is Not Altered in *Map2K7*^*+/−*^ Mice

**Fig 3.**
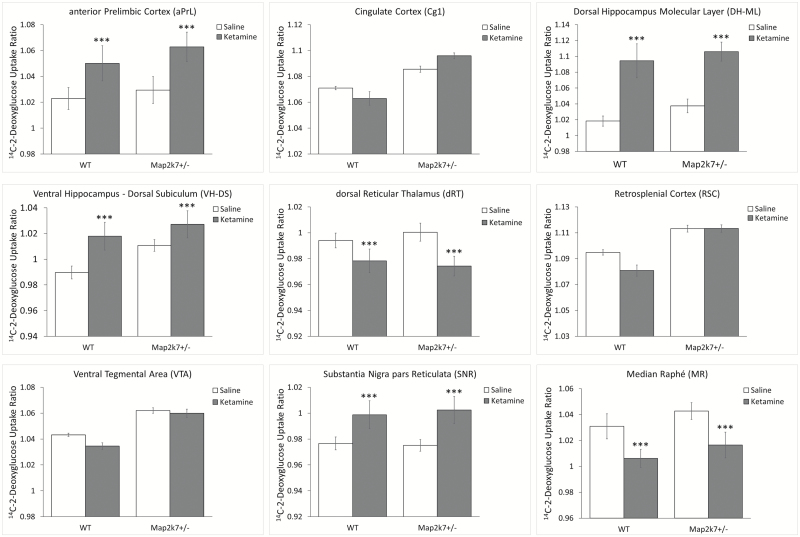
The LCGU response to ketamine is not altered in *Map2k7*^*+/−*^ mice. Data shown as mean ± standard error of the mean. ***P < .001 effect of ketamine (ANOVA).

In accordance with published data subanesthetic ketamine increased LCGU in the PFC, hippocampus and nucleus accumbens and induced hypometabolism in the reticular thalamus and selected neuromodulatory nuclei.^39,49^ We found no evidence that the response to ketamine was significantly altered in *Map2k7*^*+/−*^ mice (figure 3). This suggests that NMDA-R/glutamate system function is not significantly disrupted in *Map2k7*^+/*−*^ mice. Full data are shown in supplementary table S8.

### The Cerebral Metabolic Response to d*-*amphetamine Is Reduced in *Map2K7*^*+/−*^ Mice

In accordance with published data d*-*amphetamine increased LCGU in the thalamus, hippocampus, PFC, and basal ganglia in WT mice.^50,58^d*-*amphetamine also increased LCGU in neuromodulatory nuclei including the raphé (DR; median, MR) and locus coeruleus (LC), while decreasing LCGU in the amygdala.

**Fig 4.**
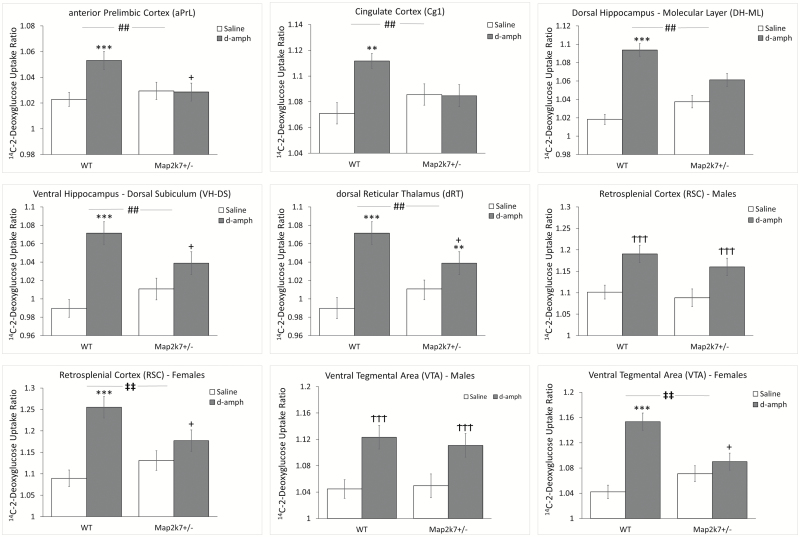
The LCGU response to d-amphetamine is attenuated in *Map2k7*^+/*−*^ mice. Data shown as mean ± standard error of the mean. ^#^*P* < .05, ^##^*P* < .01 genotype × treatment (ANOVA). **P < .01, ***P < .001 d*-*amphetamine effect within genotype (Tukey’s honestly significant difference [HSD]). ^+^*P* < .05 difference from wild-type (WT) within same treatment (Tukey’s HSD). Significant sex × genotype × treatment interactions in retrosplenial cortex (RSC) (*F*_(1,44)_=5.08, *P* = .029) and VTA (*F*_(1,44)_=5.52, *P* = .023). ^‡‡^*P* < .01 genotype × treatment interaction within sex (ANOVA). ^†††^*P*<.001 d*-*amphetamine effect within sex (ANOVA).

We found widespread evidence that the LCGU response to d*-*amphetamine was attenuated in *Map2k7*^*+/−*^ mice. This included a significantly reduced LCGU response in multiple PFC (aPrL, DLO, ventral orbital [VO], medial orbital [MO], cingulate [Cg1]), thalamic (AM, AV, MD, VM, dRT), and hippocampal (DH-ML, VH-DS) regions (figure 4). These effects were supported by significant genotype × treatment interaction in each RoI (ANOVA) and confirmed by post hoc testing (Tukey’s honestly significant difference [HSD]). For two brain regions the impact of *Map2k7* heterozygosity on the LCGU response to d*-*amphetamine was found to be more pronounced in females than in males. In this way a significant sex × genotype × treatment interaction was found in the RSC (*F*_(1,44)_ = 5.08, *P* = .029) and VTA (*F*_(1,44)_ = 5.52, *P* = .023). When each sex was analyzed separately a significant genotype × treatment interaction was identified in females (RSC, *F*_(1,25)_ = 13.52, *P* = .001; VTA, *F*_(1,25)_ = 18.73, *P* < .001) but not in males (RSC, *F*_(1,19)_ = 0.33, *P* = .574, VTA, *F*_(1,19)_ = 0.530, *P* = .476). Post hoc analysis in females confirmed that while d*-*amphetamine significantly increased LCGU in WT animals (RSC, *P* < .001; VTA, *P* < .001; Tukey’s HSD) the drug had no effect in *Map2k7*^*+/−*^ mice (RSC, *P* = .434, VTA, *P* = .919). Full data are shown in supplementary table S9.

### Male *Map2k7*^*+/−*^ Mice Show a PPI Deficit and Attenuated Responses to d*-*amphetamine

**Fig 5.**
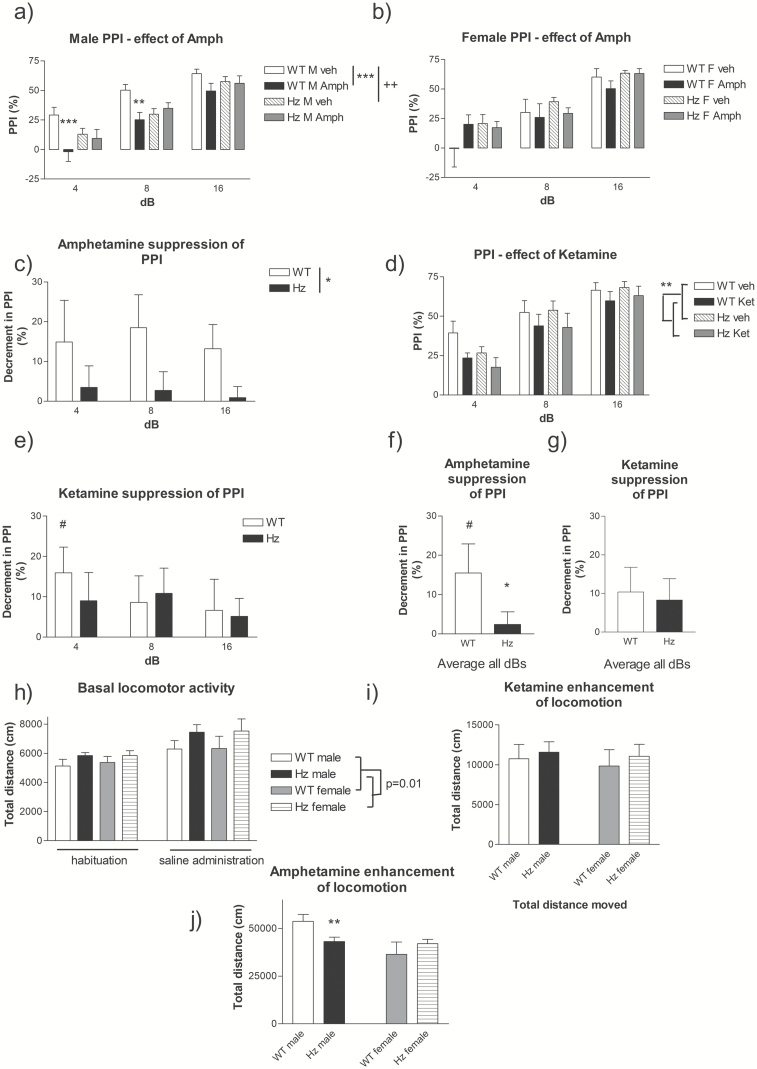
Analysis of *Map2k7*^+/*−*^ mice in behavioral assays relevant to positive symptomatology. PPI in (**a**) male and (**b**) female mice following saline or d*-*amphetamine (5 mg/kg). Males: effect of d*-*amphetamine (*F*_(1,131)_ = 14.34, *P* < .001) and genotype × d*-*amphetamine (*F*_(1,131)_ = 14.45, *P* < .001). Females: no significant effect of d*-*amphetamine or d*-*amphetamine × genotype. ** *P* < .01, *** *P* < .001 treatment effect (Fisher’s post hoc), ^++^*P* < .01 genotype effect. Mean decrement in PPI (vehicle – d*-*amphetamine) for sex-pooled data (**c**) separated stimulus dB and (**f**) pooled dBs. * *P* < .05 genotype effect ((c) ANOVA, (f) Kruskal–Wallis). (**d**, **e**, and **g**) Ketamine effects on PPI (*F*_(1,95)_ = 11.30, *P* < .001) shown as % PPI for pooled-sex mice (d) effect of ketamine (*F*_(1,95)_ = 11.05, *P* = .002), genotype × ketamine *P* = .92. **P < .01 genotype effect. Mean decrement in PPI (vehicle – ketamine) for pooled-sex mice, (e) separated stimulus dB and (g) pooled dBs. (**h**) Basal LMA during habituation (15 min) and after saline administration (30 min). *Map2k7*^*+/−*^ mice showed elevated LMA relative to wild-type (WT) (*F*_(1,39)_ = 8.3, *P* = .01). (**i**) Ketamine (20 mg/kg) induced hyperactivity in both WT and *Map2k7*^*+/−*^ mice (*F*_(1,359)_ = 54.5, *P* < .001). (**j**) d-amphetamine (3 mg/kg) also induced hyperactivity (*F*_(1,353)_ = 1763, *P* < .001, pooled sex), which was significantly attenuated in male *Map2k7*^*+/−*^ mice relative to male WTs (**P < .01, Tukey’s post hoc).

As d*-*amphetamine disrupts PPI and induces hyperlocomotion^59,60^ we assessed behavioral sensitivity to d*-*amphetamine in *Map2k7*^+/*−*^ mice. Sex-dependent effects were observed, whereby male *Map2k7*^+/*−*^ mice showed reduced baseline PPI relative to WT males, with no baseline genotype difference in females (figures 5a and 5b). d*-*amphetamine significantly reduced PPI in WT male but not WT female mice (figures 5a and 5b), limiting the detection of an attenuated d*-*amphetamine response in female *Map2k7*^*+/−*^ mice. Nevertheless, the impact of d*-*amphetamine on PPI was significantly attenuated in male *Map2k7*^+/*−*^ mice (figure 5a), and remained significant when data from both sexes was pooled (figures 5c and 5f). In parallel, d-amphetamine’s stimulatory effects on LMA was significantly attenuated in male (*F*_(1,353)_ = 1763, *P* < .001) but not female *Map2k7*^+/*−*^ mice (figure 5j).

### *Map2k7* ^*+/−*^ Mice Show Hyperlocomotion and Preserved Behavioral Responses to Ketamine

As LCGU responses to ketamine were unaltered in *Map2k7*^+/*−*^ mice (figure 3), we tested if this was evident behaviorally by characterizing ketamine’s impact on PPI and LMA.^61,62^ Consistent with brain imaging data the impact of ketamine on PPI (*F*_(1,95)_ = 111.30, *P* < .001) was not attenuated in *Map2k7*^+/*−*^ mice (*F*_(1,95)_ = 0.01, *P* = .92, figures 5d and 5e). Comparable results were obtained when measuring LMA where ketamine induced hyperactivity in both WT and *Map2k7*^+/*−*^ mice (*F*_(1,359)_ = 54.5, *P* < .001, figure 5i). Although *Map2k7*^+/*−*^ mice were significantly hyperactive relative to WT (*F*_(1,359)_ = 10.9, *P* = .001, figure 5h) there was no difference in the magnitude of the ketamine response between *Map2k7*^*+/−*^ and WT mice (*P* = .457, figure 5i).

## Discussion

*Map2k7* ^*+/−*^ mice show brain imaging endophenotypes and behavioral phenotypes relevant to ScZ. Behaviorally this includes a deficit in sensorimotor gating and hyperlocomotor activity, relevant to the positive symptoms of ScZ.^12–15^ Previous studies have identified cognitive and reward processing deficits in *Map2k7*^*+/−*^ mice, also relevant to ScZ.^3,6^ The brain metabolism and network connectivity deficits identified in *Map2k7*^*+/−*^ mice align with these behavioral deficits. Moreover, *Map2k7*^*+/−*^ mice show altered cerebral metabolism and behavioral responses to d*-*amphetamine but not ketamine, supporting monoaminergic system but not NMDA-R/glutamate system dysfunction as a consequence of *Map2k7* haploinsufficiency.

The endophenotype concept continues to evolve in psychiatric genetics.^63–65^ Key features include the following: (1) heritability and cosegregation with a disorder, or with a symptom domain that may cross diagnostic boundaries. Evidence for the association of *MAP2K7* gene variants that result in decreased *MAP2K7* expression with ScZ^3^ supports the construct validity of *Map2k7*^*+/−*^ mice. (2) The endophenotype being part of the biological disease process (eg, functional or structural brain changes) and relating to the symptoms of the disease. We discuss below the strong translational alignment between the imaging endophenotypes and behavioral deficits identified in *Map2k7*^*+/−*^ mice and those seen in ScZ. We also discuss the alignment of the imaging endophenotypes seen in these animals with their behavioral deficits.

### *Map2k7* ^*+/−*^ Mice Show ScZ-Relevant Alterations in Cerebral Metabolism and Brain Network Connectivity

The alterations in LCGU identified in *Map2k7*^*+/−*^ mice parallel those observed in ScZ. This includes DLO hypometabolism and hippocampal hypermetabolism, mirroring orbitofrontal hypometabolism^19,66^ and hippocampal/temporoparietal hypermetabolism in ScZ.^21–24^ Intriguingly, we have previously identified DLO hypometabolism in genetic^39^ and pharmacological^38^ rodent models relevant to the disorder. The alterations in functional brain network connectivity present in *Map2k7*^*+/−*^ mice also parallel those in ScZ. Although the decreased path length (figure 2) and increased regional centrality (table 1) in *Map2k7*^*+/−*^ mice contrasts with that reported in chronic ScZ,^29–31^ it does parallel the increased connectivity seen in first episode psychosis,^26^ patients with prominent auditory hallucinations^21^ and those with childhood onset ScZ.^28^ Increased global efficiency of brain network connectivity has also been reported in chronic ScZ.^67^ We also found evidence for increased PFC connectivity in *Map2k7*^*+/−*^ mice, paralleling that reported in early but not chronic ScZ and in those at high risk of developing the disorder.^25^ Increased PFC connectivity is also found after ketamine administration,^25,48,49^ a translational model relevant to ScZ, and is present in other genetic mouse models.^39^ This suggests that specific neural systems, such as the PFC, may show increased connectivity at early disease stages or in those at risk of developing ScZ, and that *Map2k7*^*+/−*^ mice may have particular translational relevance to these populations.

Alterations in inter-regional connectivity in *Map2k7*^*+/−*^ mice also have relevance to those in ScZ. This includes increased PFC-accumbens,^32,33^ reduced VTA-thalamus,^34^ reduced VTA-insular cortex,^35^ and abnormal hippocampal connectivity. Increased hippocampal–PFC connectivity in non-medicated ScZ patients^36^ and those with pronounced auditory hallucinations^33^ has been reported, as has increased default mode network connectivity in those at risk of developing psychosis.^68^ Studies have reported regional specificity in the altered hippocampal–PFC connectivity present in patients, and whether increases or decreases in connectivity are found.^36,69^ There may be a genetic basis to these divergent observations as both increased (*ZNF804A*)^70^ and decreased (*Disc1*^39^; 22q11.2^71^) hippocampal–PFC connectivity are reported in risk gene mouse models. Many of the functional connectivity alterations present in *Map2k7*^*+/−*^ mice are predictive of antipsychotic response in patients, including alterations in hippocampal–PFC,^72^ VTA–thalamus,^34^ and PFC–accumbens connectivity,^34,72^ suggesting that these may provide translational biomarkers against which the efficacy of novel antipsychotics can be tested in *Map2k7*^*+/−*^ mice.

### Brain Imaging Endophenotypes in *Map2k7*^*+/−*^ Mice Align With Their Translational Behavioral Deficits

*Map2k7* ^*+/-*^ mice show a deficit in PFC-dependent working memory^3,6^ and, in this study, hypofrontality (figure 1). As *MAP2K7* expression levels are decreased in the PFC in ScZ^3^ and *Map2k7* modulates PFC-dependent cognitive processes and metabolism, Map2k7 dysfunction may contribute to the hypofrontality^19^ and working memory deficits^73^ seen in ScZ. In addition, *Map2k7*^*+/−*^ mice show altered reward processing^6^ that may relate to their altered mesolimbic system function (figures 1 and 2). In humans, mesolimbic system connectivity is linked to trait anhedonia^74^ suggesting that the mesolimbic dysconnectivity and reward processing deficits seen in *Map2k7*^*+/−*^ mice may have relevance to the anhedonia and altered reward processing present in patients.^75,76^

We detected reduced PPI in male, but not female, *Map2k7*^+/*−*^mice. Impaired PPI is reliably observed in ScZ, may be more pronounced in male patients,^77^ and correlates with positive symptoms.^12–15^ PPI is a multisystem phenomenon, involving many brain regions including the RT, MD, PFC, and hippocampus,^78^ systems found to be dysfunctional in *Map2k7*^*+/−*^ mice.

### Altered Septal–Hippocampal Connectivity in *Map2k7*^*+/−*^ Mice May Contribute to Their Altered Response to d*-*amphetamine

*Map2k7* ^*+/−*^ mice show increased septum/DB–hippocampal connectivity (table 1, figure 2). The septum/DB regulates hippocampal activity, including the regulation of hippocampal theta waves.^79,80^ These waves are dysfunctional in ScZ, modulate hippocampal–PFC connectivity^81^ and are influenced by other ScZ risk genes.^70^ The septum also modulates VTA dopaminergic neuron activity via the hippocampus and influences the response to amphetamine.^82^ Intriguingly, we found increased hippocampal and VTA activity in *Map2k*^*+/−*^ mice (figure 1), suggesting that increased septal–hippocampal connectivity (figure 2) may drive increased hippocampal-VTA circuit activity in these animals. Previous studies have shown that lesioning^83^ or modulating activity in the septum^84^ alters the response to amphetamine. Given the key role of the septo-hippocampal pathway in PPI,^85^ and that the impact of d*-*amphetamine on hippocampal and VTA activity is attenuated in *Map2k7*^*+/−*^ mice (figure 4), dysfunction in this circuitry may contribute to the baseline PPI deficits and the attenuated impact of d*-*amphetamine on PPI in *Map2k7*^*+/−*^ mice. Intriguingly, the impact of amphetamine on cerebral metabolism and PFC dopamine release is attenuated in ScZ, mirroring the observations in *Map2k7*^*+/−*^ mice.^86,87^

Overall, the data suggest that *Map2k7*^*+/−*^ mice display a range of brain imaging and behavioral deficits that have translational relevance to ScZ. The dysfunction appears to be relevant to a broad range of ScZ symptoms, against which the efficacy of novel therapeutics can be tested. Brain imaging endophenotypes in these animals with translational relevance to positive symptoms may be particularly useful because of the paucity and ambiguity of current behavioral phenotypes for these symptoms.

## Funding

This work was supported by the Medical Research Council (award no. MR/K501335/1 to Dr Openshaw).

## Supplementary Material

sbz044_suppl_Supplementary_Figure-S1

sbz044_suppl_Supplementary_Figure-S2

sbz044_suppl_Supplementary_Table-S1

sbz044_suppl_Supplementary_Table-S2

sbz044_suppl_Supplementary_Tables-S3-S7

sbz044_suppl_Supplementary_Table-S8

sbz044_suppl_Supplementary_Table-S9

sbz044_suppl_Supplementary_Methods

## References

[1] Coffey ET Nuclear and cytosolic JNK signalling in neurons. Nat Rev Neurosci. 2014;15(5):285–299. 24739785 10.1038/nrn3729

[2] Kunde SA, Rademacher N, Tzschach A, et al Characterisation of de novo MAPK10/JNK3 truncation mutations associated with cognitive disorders in two unrelated patients. Hum Genet. 2013;132(4):461–471. 23329067 10.1007/s00439-012-1260-5

[3] Winchester CL, Ohzeki H, Vouyiouklis DA, et al Converging evidence that sequence variations in the novel candidate gene *MAP2K7* (*MKK7*) are functionally associated with schizophrenia. Hum Mol Genet. 2012;21(22):4910–4921. 22899651 10.1093/hmg/dds331

[4] Schizophrenia Working Group of the Psychiatric Genomics Consortium. Biological insights from 108 schizophrenia-associated genetic loci. Nature. 2014;511:421–427. 25056061 10.1038/nature13595 PMC4112379

[5] Morris BJ, Pratt JA Novel treatment strategies for schizophrenia from improved understanding of genetic risk. Clin Genet. 2014;86(5):401–411. 25142969 10.1111/cge.12485

[6] Openshaw RL, Thomson DM, Penninger JM, Pratt JA, Morris BJ Mice haploinsufficient for *Map2k7*, a gene involved in neurodevelopment and risk for schizophrenia, show impaired attention, a vigilance decrement deficit and unstable cognitive processing in an attentional task: impact of minocycline. Psychopharmacology (Berl). 2017;234(2):293–305. 27774567 10.1007/s00213-016-4463-y PMC5203862

[7] Carter CS, Barch DM; CNTRICS Executive Committee Imaging biomarkers for treatment development for impaired cognition: report of the sixth CNTRICS meeting: biomarkers recommended for further development. Schizophr Bull. 2012;38(1):26–33. 21914642 10.1093/schbul/sbr109 PMC3245593

[8] Millan MJ, Bales KL Towards improved animal models for evaluating social cognition and its disruption in schizophrenia: the CNTRICS initiative. Neurosci Biobehav Rev. 2013;37(9 pt B):2166–2180. 24090822 10.1016/j.neubiorev.2013.09.012

[9] Pratt J, Winchester C, Dawson N, Morris B Advancing schizophrenia drug discovery: optimizing rodent models to bridge the translational gap. Nat Rev Drug Discov. 2012;11(7):560–579. 22722532 10.1038/nrd3649

[10] Swerdlow NR, Geyer MA Using an animal model of deficient sensorimotor gating to study the pathophysiology and new treatments of schizophrenia. Schizophr Bull. 1998;24(2):285–301. 9613626 10.1093/oxfordjournals.schbul.a033326

[11] van den Buuse M Modeling the positive symptoms of schizophrenia in genetically modified mice: pharmacology and methodology aspects. Schizophr Bull. 2010;36(2):246–270. 19900963 10.1093/schbul/sbp132 PMC2833124

[12] Braff DL, Swerdlow NR, Geyer MA Symptom correlates of prepulse inhibition deficits in male schizophrenic patients. Am J Psychiatry. 1999;156(4):596–602. 10200740 10.1176/ajp.156.4.596

[13] Duncan EJ, Bollini AM, Lewison B, et al Medication status affects the relationship of symptoms to prepulse inhibition of acoustic startle in schizophrenia. Psychiatry Res. 2006;145(2-3):137–145. 17070928 10.1016/j.psychres.2006.04.006

[14] Wang ZR, Tan YL, Yang FD, et al Impaired prepulse inhibition of acoustic startle in Chinese patients with first-episode, medication-naïve schizophrenia. Chin Med J (Engl). 2013;126(3):526–531. 23422119

[15] Matsuo J, Ota M, Hori H, et al A large single ethnicity study of prepulse inhibition in schizophrenia: separate analysis by sex focusing on effect of symptoms. J Psychiatr Res. 2016;82:155–162. 27505440 10.1016/j.jpsychires.2016.07.026

[16] van den Buuse M, Wischhof L, Lee RX, Martin S, Karl T Neuregulin 1 hypomorphic mutant mice: enhanced baseline locomotor activity but normal psychotropic drug-induced hyperlocomotion and prepulse inhibition regulation. Int J Neuropsychopharmacol. 2009;12(10):1383–1393. 19400983 10.1017/S1461145709000388

[17] Clapcote SJ, Lipina TV, Millar JK, et al Behavioral phenotypes of *Disc1* missense mutations in mice. Neuron. 2007;54(3):387–402. 17481393 10.1016/j.neuron.2007.04.015

[18] Perry W, Minassian A, Henry B, Kincaid M, Young JW, Geyer MA Quantifying over-activity in bipolar and schizophrenia patients in a human open field paradigm. Psychiatry Res. 2010;178(1):84–91. 20471103 10.1016/j.psychres.2010.04.032 PMC2914139

[19] Hill K, Mann L, Laws KR, Stephenson CM, Nimmo-Smith I, McKenna PJ Hypofrontality in schizophrenia: a meta-analysis of functional imaging studies. Acta Psychiatr Scand. 2004;110(4):243–256. 15352925 10.1111/j.1600-0447.2004.00376.x

[20] Minzenberg MJ, Laird AR, Thelen S, Carter CS, Glahn DC Meta-analysis of 41 functional neuroimaging studies of executive function in schizophrenia. Arch Gen Psychiatry. 2009;66(8):811–822. 19652121 10.1001/archgenpsychiatry.2009.91 PMC2888482

[21] Silbersweig DA, Stern E, Frith C, et al A functional neuroanatomy of hallucinations in schizophrenia. Nature. 1995;378(6553):176–179. 7477318 10.1038/378176a0

[22] Epstein J, Stern E, Silbersweig D Mesolimbic activity associated with psychosis in schizophrenia. Symptom-specific PET studies. Ann N Y Acad Sci. 1999;877:562–574. 10415671 10.1111/j.1749-6632.1999.tb09289.x

[23] Shergill SS, Brammer MJ, Williams SC, Murray RM, McGuire PK Mapping auditory hallucinations in schizophrenia using functional magnetic resonance imaging. Arch Gen Psychiatry. 2000;57(11):1033–1038. 11074868 10.1001/archpsyc.57.11.1033

[24] Lieberman JA, Girgis RR, Brucato G, et al Hippocampal dysfunction in the pathophysiology of schizophrenia: a selective review and hypothesis for early detection and intervention. Mol Psychiatry. 2018;23(8):1764–1772. 29311665 10.1038/mp.2017.249 PMC6037569

[25] Anticevic A, Corlett PR, Cole MW, et al N-methyl-D-aspartate receptor antagonist effects on prefrontal cortical connectivity better model early than chronic schizophrenia. Biol Psychiatry. 2015;77(6):569–580. 25281999 10.1016/j.biopsych.2014.07.022

[26] Wang H, Zhang B, Zeng B, et al Association between catechol-O-methyltransferase genetic variation and functional connectivity in patients with first-episode schizophrenia. Schizophr Res. 2018;199:214–220. 29730044 10.1016/j.schres.2018.04.023

[27] Chen C, Wang HL, Wu SH, et al Abnormal degree centrality of bilateral putamen and left superior frontal gyrus in schizophrenia with auditory hallucinations: a resting-state functional magnetic resonance imaging study. Chin Med J (Engl). 2015;128(23):3178–3184. 26612293 10.4103/0366-6999.170269 PMC4794878

[28] Alexander-Bloch AF, Gogtay N, Meunier D, et al Disrupted modularity and local connectivity of brain functional networks in childhood-onset schizophrenia. Front Syst Neurosci. 2010;4:147. 21031030 10.3389/fnsys.2010.00147 PMC2965020

[29] Micheloyannis S, Pachou E, Stam CJ, et al Small-world networks and disturbed functional connectivity in schizophrenia. Schizophr Res. 2006;87(1-3):60–66. 16875801 10.1016/j.schres.2006.06.028

[30] Liu Y, Liang M, Zhou Y, et al Disrupted small-world networks in schizophrenia. Brain. 2008;131(pt 4):945–961. 18299296 10.1093/brain/awn018

[31] Rubinov M, Bullmore E Schizophrenia and abnormal brain network hubs. Dialogues Clin Neurosci. 2013;15(3):339–349. 24174905 10.31887/DCNS.2013.15.3/mrubinov PMC3811105

[32] Rolland B, Amad A, Poulet E, et al Resting-state functional connectivity of the nucleus accumbens in auditory and visual hallucinations in schizophrenia. Schizophr Bull. 2015;41(1):291–299. 25053649 10.1093/schbul/sbu097 PMC4266295

[33] Amad A, Cachia A, Gorwood P, et al The multimodal connectivity of the hippocampal complex in auditory and visual hallucinations. Mol Psychiatry. 2014;19(2):184–191. 23318999 10.1038/mp.2012.181

[34] Hadley JA, Nenert R, Kraguljac NV, et al Ventral tegmental area/midbrain functional connectivity and response to antipsychotic medication in schizophrenia. Neuropsychopharmacol. 2014;39(4):1020–1030. 10.1038/npp.2013.305 PMC3924537 24165885

[35] Giordano GM, Stanziano M, Papa M, et al Functional connectivity of the ventral tegmental area and avolition in subjects with schizophrenia: a resting state functional MRI study. Eur Neuropsychopharmacol. 2018;28(5):589–602. 29653743 10.1016/j.euroneuro.2018.03.013

[36] Kraguljac NV, White DM, Hadley N, et al Aberrant hippocampal connectivity in unmedicated patients with schizophrenia and effects of antipsychotic medication: a longitudinal resting state functional MRI study. Schizophr Bull. 2016;42(4):1046–1055. 26873890 10.1093/schbul/sbv228 PMC4903060

[37] Cochran SM, Kennedy M, McKerchar CE, Steward LJ, Pratt JA, Morris BJ Induction of metabolic hypofunction and neurochemical deficits after chronic intermittent exposure to phencyclidine: differential modulation by antipsychotic drugs. Neuropsychopharmacol. 2003;28(2):265–275. 10.1038/sj.npp.1300031 12589379

[38] Dawson N, Thompson RJ, McVie A, Thomson DM, Morris BJ, Pratt JA Modafinil reverses phencyclidine-induced deficits in cognitive flexibility, cerebral metabolism, and functional brain connectivity. Schizophr Bull. 2012;38(3):457–474. 20810469 10.1093/schbul/sbq090 PMC3329989

[39] Dawson N, Kurihara M, Thomson DM, et al Altered functional brain network connectivity and glutamate system function in transgenic mice expressing truncated *Disrupted-in-schizophrenia 1*. Transl Psychiatry. 2015;5:e569. 25989143 10.1038/tp.2015.60 PMC4471291

[40] Dawson N, Xiao X, McDonald M, Higham DJ, Morris BJ, Pratt JA Sustained NMDA receptor hypofunction induces compromised neural systems integration and schizophrenia-like alterations in functional brain networks. Cereb Cortex. 2014;24(2):452–464. 23081884 10.1093/cercor/bhs322

[41] Dawson N, Morris BJ, Pratt JA Functional brain connectivity phenotypes for schizophrenia drug discovery. J Psychopharmacol. 2015;29(2):169–177. 25567554 10.1177/0269881114563635

[42] Maletic V, Eramo A, Gwin K, Offord SJ, Duffy RA The role of norepinephrine and its α-adrenergic receptors in the pathophysiology and treatment of major depressive disorder and schizophrenia: a systematic review. Front Psychiatry. 2017;8:42. 28367128 10.3389/fpsyt.2017.00042 PMC5355451

[43] Selvaraj S, Arnone D, Cappai A, Howes O Alterations in the serotonin system in schizophrenia: a systematic review and meta-analysis of postmortem and molecular imaging studies. Neurosci Biobehav Rev. 2014;45:233–245. 24971825 10.1016/j.neubiorev.2014.06.005

[44] Howes O, McCutcheon R, Stone J Glutamate and dopamine in schizophrenia: an update for the 21st century. J Psychopharmacol. 2015;29(2):97–115. 25586400 10.1177/0269881114563634 PMC4902122

[45] Dauvermann MR, Lee G, Dawson N Glutamatergic regulation of cognition and functional brain connectivity: insights from pharmacological, genetic and translational schizophrenia research. Br J Pharmacol. 2017;174(19):3136–3160. 28626937 10.1111/bph.13919 PMC5595770

[46] Dahoun T, Trossbach SV, Brandon NJ, Korth C, Howes OD The impact of *Disrupted-in-Schizophrenia 1* (*DISC1*) on the dopaminergic system: a systematic review. Transl Psychiatry. 2017;7(1):e1015. 28140405 10.1038/tp.2016.282 PMC5299392

[47] Sasaki T, Wada T, Kishimoto H, et al The stress kinase mitogen-activated protein kinase kinase (MKK)7 is a negative regulator of antigen receptor and growth factor receptor-induced proliferation in hematopoietic cells. J Exp Med. 2001;194(6):757–768. 11560992 10.1084/jem.194.6.757 PMC2195963

[48] Dawson N, McDonald M, Higham DJ, Morris BJ, Pratt JA Subanesthetic ketamine treatment promotes abnormal interactions between neural subsystems and alters the properties of functional brain networks. Neuropsychopharmacol. 2014;39(7):1786–1798. 10.1038/npp.2014.26 PMC4023152 24492765

[49] Dawson N, Morris BJ, Pratt JA Subanaesthetic ketamine treatment alters prefrontal cortex connectivity with thalamus and ascending subcortical systems. Schizophr Bull. 2013;39(2):366–377. 22114100 10.1093/schbul/sbr144 PMC3576175

[50] Miyamoto S, Leipzig JN, Lieberman JA, Duncan GE Effects of ketamine, MK-801, and amphetamine on regional brain 2-deoxyglucose uptake in freely moving mice. Neuropsychopharmacol. 2000;22(4):400–412. 10.1016/S0893-133X(99)00127-X 10700659

[51] Mevik B-H, Wehrens R The pls package: principal component and partial least squares regression in R. J. Stat. Software. 2007;18:1– 23.

[52] Wold S, Sjostrom M, Eriksson L PLS-regression: a basic tool of chemometrics. Chemometr Intell Lab. 2001;58:109–130.

[53] Kouskou M, Thomson DM, Brett RR, et al Disruption of the *Zdhhc9* intellectual disability gene leads to behavioural abnormalities in a mouse model. Exp Neurol. 2018;308:35–46. 29944857 10.1016/j.expneurol.2018.06.014 PMC6104741

[54] van den Buuse M, van Driel IR, Samuelson LC, Pijnappel M, Martin S Reduced effects of amphetamine on prepulse inhibition of startle in gastrin-deficient mice. Neurosci Lett. 2005;373(3):237–242. 15619550 10.1016/j.neulet.2004.10.013

[55] Zanos P, Piantadosi SC, Wu HQ, et al The prodrug 4-chlorokynurenine causes ketamine-like antidepressant effects, but not side effects, by NMDA/GlycineB-site inhibition. J Pharmacol Exp Ther. 2015;355(1):76–85. 26265321 10.1124/jpet.115.225664 PMC4576668

[56] Melander T, Staines WA, Hökfelt T, et al Galanin-like immunoreactivity in cholinergic neurons of the septum-basal forebrain complex projecting to the hippocampus of the rat. Brain Res. 1985;360(1–2):130–138. 2416401 10.1016/0006-8993(85)91228-4

[57] O’Mara S The subiculum: what it does, what it might do, and what neuroanatomy has yet to tell us. J Anat. 2005;207(3):271–282. 16185252 10.1111/j.1469-7580.2005.00446.x PMC1571536

[58] Orzi F, Dow-Edwards D, Jehle J, Kennedy C, Sokoloff L Comparative effects of acute and chronic administration of amphetamine on local cerebral glucose utilization in the conscious rat. J Cereb Blood Flow Metab. 1983;3(2):154–160. 6841462 10.1038/jcbfm.1983.22

[59] Moy SS, Perez A, Koller BH, Duncan GE Amphetamine-induced disruption of prepulse inhibition in mice with reduced NMDA receptor function. Brain Res. 2006;1089(1):186–194. 16638606 10.1016/j.brainres.2006.03.073

[60] McNamara RK, Logue A, Stanford K, Xu M, Zhang J, Richtand NM Dose-response analysis of locomotor activity and stereotypy in dopamine D3 receptor mutant mice following acute amphetamine. Synapse 2006;60(5):399–405. 16856172 10.1002/syn.20315 PMC1815379

[61] Hetzler BE, Wautlet BS Ketamine-induced locomotion in rats in an open-field. Pharmacol Biochem Behav. 1985;22(4):653–655. 3991775 10.1016/0091-3057(85)90291-6

[62] Irifune M, Shimizu T, Nomoto M Ketamine-induced hyperlocomotion associated with alteration of presynaptic components of dopamine neurons in the nucleus accumbens of mice. Pharmacol Biochem Behav. 1991;40(2):399–407. 1805243 10.1016/0091-3057(91)90571-i

[63] Flint J, Munafò MR The endophenotype concept in psychiatric genetics. Psychol Med. 2007;37(2):163–180. 16978446 10.1017/S0033291706008750 PMC2829981

[64] Krol A, Wimmer RD, Halassa MM, Feng G Thalamic reticular dysfunction as a circuit endophenotype in neurodevelopmental disorders. Neuron. 2018;98(2):282–295. 29673480 10.1016/j.neuron.2018.03.021 PMC6886707

[65] Rosen AM, Spellman T, Gordon JA Electrophysiological endophenotypes in rodent models of schizophrenia and psychosis. Biol Psychiatry. 2015;77(12):1041–1049. 25910423 10.1016/j.biopsych.2015.03.021 PMC4444383

[66] Glahn DC, Ragland JD, Abramoff A, et al Beyond hypofrontality: a quantitative meta-analysis of functional neuroimaging studies of working memory in schizophrenia. Hum Brain Mapp. 2005;25(1):60–69. 15846819 10.1002/hbm.20138 PMC6871703

[67] Lynall ME, Bassett DS, Kerwin R, et al Functional connectivity and brain networks in schizophrenia. J Neurosci. 2010;30(28):9477–9487. 20631176 10.1523/JNEUROSCI.0333-10.2010 PMC2914251

[68] Shim G, Oh JS, Jung WH, et al Altered resting-state connectivity in subjects at ultra-high risk for psychosis: an fMRI study. Behav Brain Funct. 2010;6:58. 20932348 10.1186/1744-9081-6-58 PMC2959003

[69] Samudra N, Ivleva EI, Hubbard NA, et al Alterations in hippocampal connectivity across the psychosis dimension. Psychiatry Res. 2015;233(2):148–157. 26123450 10.1016/j.pscychresns.2015.06.004 PMC4784701

[70] Cousijn H, Tunbridge EM, Rolinski M, et al Modulation of hippocampal theta and hippocampal-prefrontal cortex function by a schizophrenia risk gene. Hum Brain Mapp. 2015;36(6):2387–2395. 25757652 10.1002/hbm.22778 PMC4672713

[71] Sigurdsson T, Stark KL, Karayiorgou M, Gogos JA, Gordon JA Impaired hippocampal-prefrontal synchrony in a genetic mouse model of schizophrenia. Nature. 2010;464(7289):763–767. 20360742 10.1038/nature08855 PMC2864584

[72] Bolding MS, White DM, Hadley JA, Weiler M, Holcomb HH, Lahti AC Antipsychotic drugs alter functional connectivity between the medial frontal cortex, hippocampus, and nucleus accumbens as measured by H_2_^15^O PET. Front Psychiatry. 2012;3:105. 23230425 10.3389/fpsyt.2012.00105 PMC3515723

[73] Senkowski D, Gallinat J Dysfunctional prefrontal gamma-band oscillations reflect working memory and other cognitive deficits in schizophrenia. Biol Psychiatry. 2015;77(12):1010–1019. 25847179 10.1016/j.biopsych.2015.02.034

[74] Keller J, Young CB, Kelley E, Prater K, Levitin DJ, Menon V Trait anhedonia is associated with reduced reactivity and connectivity of mesolimbic and paralimbic reward pathways. J Psychiatr Res. 2013;47(10):1319–1328. 23791396 10.1016/j.jpsychires.2013.05.015

[75] Chase HW, Loriemi P, Wensing T, Eickhoff SB, Nickl-Jockschat T Meta-analytic evidence for altered mesolimbic responses to reward in schizophrenia. Hum Brain Mapp. 2018;39(7):2917–2928. 29573046 10.1002/hbm.24049 PMC6866586

[76] Strauss GP, Waltz JA, Gold JM A review of reward processing and motivational impairment in schizophrenia. Schizophr Bull. 2014;40 (suppl 2):S107–S116. 24375459 10.1093/schbul/sbt197 PMC3934394

[77] Kumari V, Aasen I, Sharma T Sex differences in prepulse inhibition deficits in chronic schizophrenia. Schizophr Res. 2004;69(2-3):219–235. 15469195 10.1016/j.schres.2003.09.010

[78] Kohl S, Heekeren K, Klosterkötter J, Kuhn J Prepulse inhibition in psychiatric disorders–apart from schizophrenia. J Psychiatr Res. 2013;47(4):445–452. 23287742 10.1016/j.jpsychires.2012.11.018

[79] Vandecasteele M, Varga V, Berényi A, et al Optogenetic activation of septal cholinergic neurons suppresses sharp wave ripples and enhances theta oscillations in the hippocampus. Proc Natl Acad Sci USA. 2014;111(37): 13535–13540. 25197052 10.1073/pnas.1411233111 PMC4169920

[80] Papouin T, Dunphy JM, Tolman M, Dineley KT, Haydon PG Septal cholinergic neuromodulation tunes the astrocyte-dependent gating of hippocampal NMDA receptors to wakefulness. Neuron. 2017;94(4):840–854.e7. 28479102 10.1016/j.neuron.2017.04.021 PMC5484087

[81] Uhlhaas PJ, Singer W Abnormal neural oscillations and synchrony in schizophrenia. Nat Rev Neurosci. 2010;11(2):100–113. 20087360 10.1038/nrn2774

[82] Lisman JE, Grace AA The hippocampal-VTA loop: controlling the entry of information into long-term memory. Neuron. 2005;46(5):703–713. 15924857 10.1016/j.neuron.2005.05.002

[83] Lecourtier L, de Vasconcelos AP, Cosquer B, Cassel JC Combined lesions of GABAergic and cholinergic septal neurons increase locomotor activity and potentiate the locomotor response to amphetamine. Behav Brain Res. 2010;213(2):175–182. 20450937 10.1016/j.bbr.2010.04.050

[84] Bortz DM, Grace AA Medial septum differentially regulates dopamine neuron activity in the rat ventral tegmental area and substantia nigra via distinct pathways. Neuropsychopharmacol. 2018;43(10):2093–2100. 10.1038/s41386-018-0048-2 PMC6098086 29654260

[85] Koch M The septohippocampal system is involved in prepulse inhibition of the acoustic startle response in rats. Behav Neurosci. 1996;110(3):468–477. 8888992 10.1037//0735-7044.110.3.468

[86] Wolkin A, Angrist B, Wolf A, et al Effects of amphetamine on local cerebral metabolism in normal and schizophrenic subjects as determined by positron emission tomography. Psychopharmacology (Berl). 1987;92(2):241–246. 3110848 10.1007/BF00177923

[87] Slifstein M, van de Giessen E, Van Snellenberg J, et al Deficits in prefrontal cortical and extrastriatal dopamine release in schizophrenia: a positron emission tomographic functional magnetic resonance imaging study. JAMA Psychiatry. 2015;72(4):316–324. 25651194 10.1001/jamapsychiatry.2014.2414 PMC4768742

