# Supplementary material for: Map2k7 Haploinsufficiency Induces Brain Imaging Endophenotypes and Behavioral Phenotypes Relevant to Schizophrenia
Source: Schizophr Bull. 2019 Jun 20;46(1):211–23. doi: 10.1093/schbul/sbz044 (PMC6942167; doi:10.1093/schbul/sbz044)
Supplement: sbz044_suppl_Supplementary_Figure-S1 [file sbz044_suppl_supplementary_figure-s1.docx]

**Supplemental Material: Figure S1**

Genetic association of MAP2K7 pathway with schizophrenia


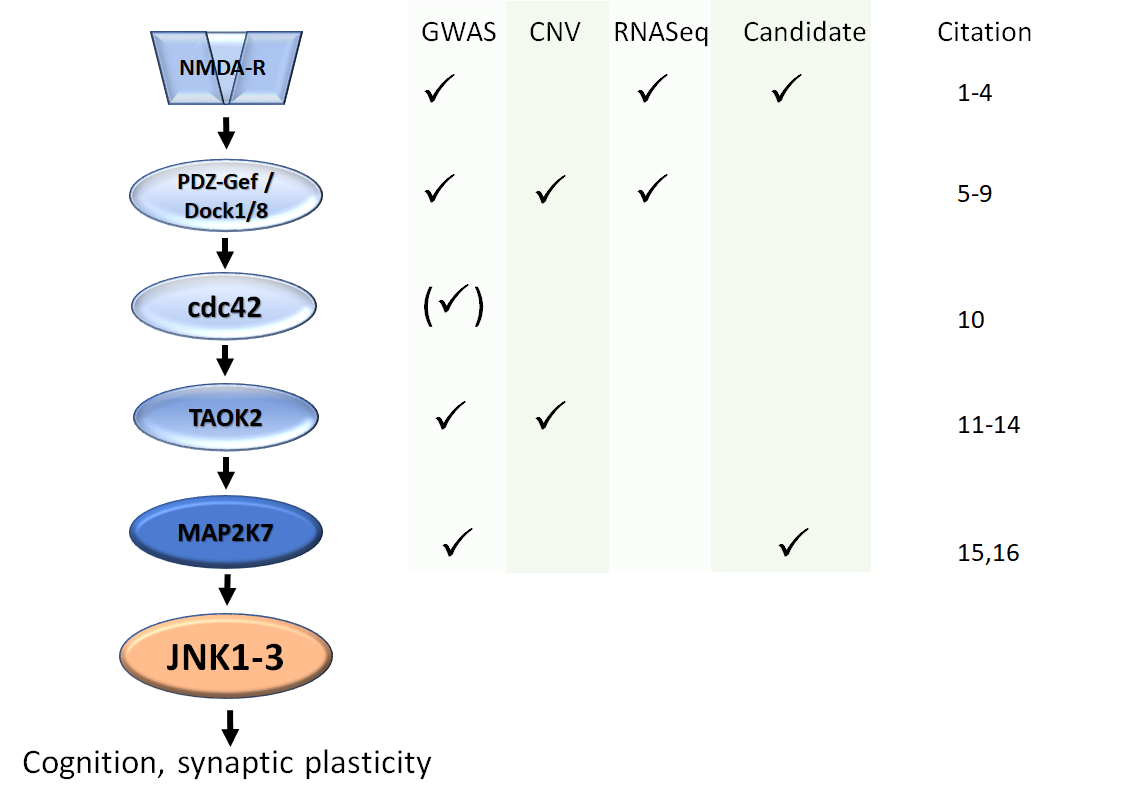


**References:**

1. Fromer M, Pocklington AJ, Kavanagh DH *et al*. De novo mutations in schizophrenia implicate synaptic networks. *Nature* 2014;506:179-184.

2. Li D, He L. Association study between the NMDA receptor 2B subunit gene (*GRIN2B*) and schizophrenia: A HuGE review and meta-analysis. *Genet Med* 2007;9:4-8.

3. Zhao X, Li H, Shi Y *et al.* Significant association between the genetic variations in the 5′ end of the N-Methyl-D-Aspartate receptor subunit gene *GRIN1* and schizophrenia. *Biol Psychiatry* 2006;59:747-753.

4. Awadalla P, Gauthier J, Myers RA *et al*. Direct measure of the de novo mutation rate in autism and schizophrenia cohorts. *Am J Hum Genet* 2010;87:316-324

5. Glessner JT, Reilly MP, Kim CE *et al*. Strong synaptic transmission impact by copy number variations in schizophrenia. *Proc Natl Acad Sci USA* 2010;107:10584-10589.

6. Xu B, Woodroffe A, Rodriguez-Murillo L *et al*. Elucidating the genetic architecture of familial schizophrenia using rare copy number variant and linkage scans. *Proc Natl Acad Sci USA* 2009: 106: 16746-16751.

7. Xu B, Ionita-Laza I, Roos JL *et al*. De novo gene mutations highlight patterns of genetic and neural complexity in schizophrenia. *Nat Genet* 2012: 44: 1365-1369

8. Neuropsychiatric Genomics Working Group. Copy number variation meta-analysis reveals a novel duplication at 9p24 associated with multiple neurodevelopmental disorders. *Genome Med* 2017;9:106.

9. Kushima I, Aleksic B, *et al*. Comparative analyses of copy-number variation in autism spectrum disorder and schizophrenia reveal etiological overlap and biological insights. *Cell Rep* 2018 11;24:2838-2856.

10. Gilks WP, Hill M, Gill M *et al*. Functional investigation of a schizophrenia GWAS signal at the CDC42 gene. *World J Biol Psychiatry* 2012; 13:550-554.

11. Zheng X, Bei J-X, Xu H *et al.* The association between rare large duplication of 16p11.2 and schizophrenia in the Singaporean Chinese population. *Schizophr Res* 2013;146:368-369.

12. McCarthy SE, Makarov V, Kirov G *et al*. Microduplications of 16p11.2 are associated with schizophrenia. *Nat Genet* 2009:41:1223-1227.

13. Addington AM, Rapoport JL. The genetics of childhood-onset schizophrenia: when madness strikes the prepubescent. *Current Psychiatr Rep* 2009:11:156-161.

14. Steinberg S, de Jong S, Mattheisen M *et al*. Common variant at 16p11.2 conferring risk of psychosis. *Mol Psychiatry* 2012;19:108-114.

15. Winchester CL, Ohzeki H, Vouyiouklis DA *et al*. Converging evidence that sequence variations in the novel candidate gene *MAP2K7* (*MKK7*) are functionally associated with schizophrenia. *Hum Mol Genet* 2012:21:4910-4921.

16. Lin WY, Chen WJ, Liu CM, Hwu HG, McCarroll SA, Glatt SJ, Tsuang MT. Adaptive combination of Bayes factors as a powerful method for the joint analysis of rare and common variants. *Sci Rep* 2017;7:13858.
