# Supplementary material for: Map2k7 Haploinsufficiency Induces Brain Imaging Endophenotypes and Behavioral Phenotypes Relevant to Schizophrenia
Source: Schizophr Bull. 2019 Jun 20;46(1):211–23. doi: 10.1093/schbul/sbz044 (PMC6942167; doi:10.1093/schbul/sbz044)
Supplement: sbz044_suppl_Supplementary_Figure-S2 [file sbz044_suppl_supplementary_figure-s2.docx]

**Figure S2. Altered regional connectivity in *Map2k7^+/-^* mice**

Increases in Regional Connectivity

Decreases in Regional Connectivity

Heatmaps showing altered inter-regional connectivity in *Map2k7^+/-^* mice. Red denotes gained (VIP 95% CI >0.8 in *Mapk7^+/-^* , <0.8 in WT and *z*-score difference >1.96) and blue denotes lost (VIP 95% CI >0.8 in WT, <0.8 in *Map2k7^+/-^* and *z*-score difference <-1.96) connectivity in *Map2k7^+/-^* mice.
