# Supplementary material for: Map2k7 Haploinsufficiency Induces Brain Imaging Endophenotypes and Behavioral Phenotypes Relevant to Schizophrenia
Source: Schizophr Bull. 2019 Jun 20;46(1):211–23. doi: 10.1093/schbul/sbz044 (PMC6942167; doi:10.1093/schbul/sbz044)
Supplement: sbz044_suppl_Supplementary_Methods [file sbz044_suppl_supplementary_methods.docx]

**Supplementary Methods: Network and connectivity analysis of ^14^C-2-deoxyglucose functional brain imaging data**

*Section A: Network Analysis of ^14^C-2-deoxyglucose brain imaging data*

**Inter-Regional Correlations and Functional Brain Networks**

The inter-regional Pearson’s correlation coefficient was used as the metric of the functional association between brain regions generated from the ^14^C-2-deoxyglucose uptake ratios for each brain region of interest (RoI) across all animals within the same experimental group (i.e., either saline-treated wild-type (WT) or saline-treated *Map2k7^+/-^* mice). These correlations were then Fisher *z*-transformed to give the correlation data a more normal distribution. This resulted in a pair of {58 x 58} correlation matrices, each within-group matrix representing the specific association strength between each of the 1653 possible pairs of regions. From each correlation matrix (R), we derived a binary adjacency matrix (A) where the functional connection between two regions (a*^i,j^* element) was zero if the correlation coefficient was lower than the defined threshold (p_|_*_i,j_*_|_<T) and unity if the coefficient was greater or equal to the defined threshold (p_|_*_i,j_*_|_≥T). The adjacency matrix can also be represented as an undirected graph, G, where a line (edge) represents the functional interaction between two brain regions (graph nodes) if the correlation coefficient exceeds the threshold.

**Network Analysis**

Brain network architecture was characterized at the global and regional scales using network science algorithms. Global network architecture was quantified in terms of the mean degree (<*k*>), average path length (L_p_), and mean clustering co-efficient (C_p_), as previously described^1,2^

and outlined in detail below. Regional properties were defined in terms of degree (*k_i_*), betweenness (B*_c_*), closeness (C*_c_*) and Eigenvector (E*_c_*) centrality. Global and regional metrics were determined on the binary adjacency matrices generated over a range of correlation thresholds (Pearson’s r, T=0.35–0.45 and Fisher’s z, T=0.37–0.48) that were selected on the basis that the maximum threshold utilized yielded fully connected networks in each experimental group, similar to the approach utilized in previous reports.^1,3,4^ All brain network analysis was completed using the igraph package^5^ in R.^6^

**Global Brain Network Architecture**

The degree of a node (*k*) is simply the number of edges that connect that node to the network, so highly connected nodes have a high degree. The mean degree (<*k*>, equation 1) is the average number of edges across all nodes. A sparse network therefore has a low mean degree.

 (1)

The minimum path length between two nodes in a graph (L_i,j_) is the lowest number of edges that must be traversed to make a connection between them. If two nodes are immediate neighbours, directly connected by a single edge, then L_i,j_=1. The average path length (L_p_, equation 2), or

average L_i,j_ across all possible node pairs, is the average number of steps along all the shortest paths across the network. This provides a measure of global network efficiency, where networks with a low average path length are more efficient for information transfer.

 (2)

The clustering coefficient of node *i* (C_i_) is the ratio of the number of edges between connected neighbours of that node relative to the maximum possible number of connections between them. This provides an indication of how well connected the neighbourhood of a node is. The mean clustering coefficient (C_p_, equation 3) is the average clustering coefficient of all of the nodes in the network, which provides a measure of the local density or cliquishness of the network. A high mean clustering coefficient suggests high local clustering and so efficient local information transfer.

 (3)

The significance of the alterations in the global network properties seen between saline-treated WT and *Map2K7^+/-^* mice was determined by comparison of the real difference in each measure with that of networks generated from 5000 random permutations of the raw data at each correlation threshold (55,000 total random permutations). Significance was set at p<0.05 and was determined from the average p-value across the entire correlation threshold range analyzed.

**Regional Centrality and Hub Brain Region Identification**

In this study, we consider node centrality as determined by degree (k*_i_*), betweenness (B*_c_*), closeness (C*_c_*) and Eigenvector (E*_c_*) centrality. Degree centrality (k*_i_*) simply measures the number of connections of a given node. Betweenness centrality (B*_c_*, equation 4) is based upon how many network short paths go through a given node, defined by:

 (4)

Here, st denotes the number of shortest paths from node s to node t, and st(*i*) denotes the number of these that involve node *i*.

Closeness centrality (C*_c_*, equation 5) is based upon the mean geodesic distance of a node to all other reachable nodes in the network, given by:

 (5)

Here, dG(*i,t*) denotes the geodesic between nodes *i* and *t*.

Eigenvector centrality (E_c_, or x_v_ in equation 6) gives an indication of how influential a node is in the context of all other nodes that are connected in the network, with nodes that are themselves connected to many other influential nodes a high score, given by:


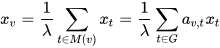
 (6)

With *M*(*v*) being the set of neighbours of the node of interest (*v*).

A brain region (or node) that is considered to be an important hub region in the network has a high degree, betweenness, closeness or eigenvector centrality. In this study, the z-score difference in each centrality measure, and for each node, in *Map2k7^+/-^* mice relative to WT animals was determined, with the centrality measures in the network of each experimental group first calibrated to that in 11,000 random Erdös-Rényi networks. Thus, positive z-score indicated an increase in centrality whereas negative z-scores indicate a decrease in centrality in the brain networks of *Map2k7^+/-^* mice relative to WT controls. For each centrality measure the significance of the difference between the experimental groups was determined by comparison to that seen in 55,000 random permutations of the real data, with significance set at p<0.05. In addition, a composite standardised z-score was calculated across all centrality measures, with a composite z-score >1.96 or <-1.96 considered to be significant between the two experimental groups.

*Section B: Application of PLSR to 2-deoxyglucose brain imaging data*

Partial least squares (also known as projection to latent structures) regression (PLSR) is a method for relating two data matrices, **X** and **Y**, by a multivariate linear regression model and is particularly suited to analysing functional connectivity in the brain as it can model the influence of multiple, collinear “predictor” (X) variables upon a given dependent (Y) variable (For a detailed over-view of PLSR see Wold *et al*., (1995)^7^ and Wold *et al*., (2001)^8^). In this way PLSR can model the functional connectivity between a “seed” brain region (dependent variable) and all of the other brain regions analysed (“explanatory” variables). In this study data were analysed using the PLS package^9^ in R. Each PLSR model is generated from a set of *N* observations (experimental cases) with *K* X-variables (“explanatory” brain regions) and *M* Y-variables (“seed” regions). These data form two matrices **X** and **Y** of dimensions (*N* x *K)* and (*N* x *M).* The linear PLSR model identifies “new” variables which are estimates of latent variables (LV’s) present within the data. In PLSR these variables are known as X-scores and are denoted by **t***a* (*a* = 1,2…,*A)*. These scores are predictors of **Y** (Equation 10) and also model **X** (Equation 8) as these matrices are modelled, at least in part, by the same LV’s. These X-scores are estimated as linear combinations of the original variables **x**_k_ with the “weight” coefficients, *w*_ka_* (a = 1,2…,*A*) (Equation 7).

 (7)

The X-scores (**t**a’s) are multiplied by the loadings *P_ak_* so that the X-residuals (*e_ik_*) are small (Equation 8).

 (8)

The corresponding Y-scores (**u**_a_) are multiplied by the weights c_am_ so that the residuals *g*_im_ are also small (Equation 9).

 (9)

In this way the X-scores are good predictors of Y (Equation 10).

 (10)

Equations (7) and (10) can be re-written as a multiple regression model (Equation 11)

 (11)

After each component, *a*, the **X**-matrix is “deflated” by subtracting *t*_ia_P_ka_* from *x_ik_* (**t**_a_ **p'**_a_ from **X**). This makes the PLSR model alternatively be expressed as weights **w**_a_ referring to the residuals after previous dimension, **E** _a-1_, rather than relating to the X-variables themselves. In the PLSR algorithm the first weight vector (**w**_1_) is the first eigenvector of the combined variance-covariance matrix, **X'YY'X**, and the following weight vectors (component *a*) are the eigenvectors to the deflated versions of the same matrix (**Z'_a_YYZ'_a_** where **Z_a_** = **Z** _a-1_ **– T** _a-1_ **P'**_a-1_). In the same way the first score vector (**t**1) is an eigenvector to **XX'YY'** and later X-score vectors (**t**_a_) are eigenvectors of **ZaZ'aYY**.

The PLSR model may be interpreted as forming new x-variables (LV estimates), **t**_a_, as linear combinations of the original x’s and then using these new *t* variables as predictors of Y, where only as many new *t*’s are formed as are predictively significant. In the model the scores, **t** and **u**, contain the information about the objects and their similarities/dissimilarities in the given model. The weights, **w**_a_ (or **w**_a_*) and **c**_a_ give information about how the variables combine to from the quantitative relationship between **X** and **Y**. Therefore, these weights are essential in understanding which of the X-variables are important (numerically large **w**_a_) and which provide the same information (similar profiles of **w**_a_ values) in the model.

It is necessary to determine the appropriate number of components, *A,* in the PLSR model to prevent over-fitting the model to the data and losing its predictive power. Therefore, cross-validation (CV) is used to ascertain the predictive validity of each of the PLSR model with a given number of components. In this study CV was completed on the “leave-one-out” basis, where one case from the experimental group is left out, the PLSR model is developed and then the predictive validity of the model assessed on the left out case. This procedure is repeated *n* times, with each case used as the validation case in turn. After developing each model the difference between the actual and predicted Y-values are calculated for the deleted data. The sum of squares for these differences is computed and collected from all the parallel models to calculate the predictive residual sum of squares (PRESS) statistic and the root mean square error of prediction (RMSEP) measures, which estimates the predictive validity of the model.

In PLSR modelling the relative importance of a variable in modelling Y is reflected by the PLS-regression value, *b_mk_*. However, a variable may also be important in modelling X, and is identified through a large loading value *P_ak_*. A summary of the importance of an x-variable (brain region) for both Y and X is given by the variable importance for the projection (VIP) statistic. In this way, in our models, the VIP can be considered to reflect the functional coupling between the defined “seed” brain region and the other brain regions of interest. The VIP is calculated from the weighted sum of squares of the PLS-weights, **w***_ak_, with the weights calculated from the amount of Y-variance of each PLSR component, *a*. VIP values <0.8 are considered to make a small contribution, 0.8-1 a moderate contribution and values >1 a large contribution to determining the values in the X and Y matrices.^8^ For each experimental group and “seed” region, the VIP statistic for each brain region (x-variable) was determined. Through a jack-knifing (leave-one-out) procedure the standard deviation (SD) of each of the VIP statistics was estimated, and subsequently used to determine the 95% confidence interval (CI) of the VIP statistic.^10^ In this study a significant predictive functional connection between brain regions was only considered to exist if the 95% CI of the VIP statistic exceeded the 0.8 threshold. Genotype-induced alterations in the VIP statistic were statistically determined through calculation of the standardized z-score between experimental groups, with z>1.96 or z<-1.96 considered to be significant.

References

1. Dawson N, Kurihara M, Thomson DM, *et al*. Altered functional brain network connectivity and glutamate system function in transgenic mice expressing truncated *Disrupted in Schizophrenia 1*. *Transl Psychiatry* 2015;5:e569.
2. Dawson N, Xiao X, McDonald M, Higham DJ, Morris BJ, Pratt JA. Sustained NMDA receptor hypofunction induces compromised neural systems integration and schizophrenia-like alterations in functional brain networks. *Cerebral Cortex* 2014; 24:452-464.
3. Liu Y, Liang M, Zhou Y, He Y, Hao Y, Song M. Disrupted small-world networks in schizophrenia. *Brain* 2008;131:945-961.
4. Micheloyannis S, Pachou E, Stam CJ, Breakspear M, Bitsios P, Vourkas M, Erimaki S, Zervakis M. Small world networks and disturbed functional connectivity in schizophrenia. *Schizophrenia Research* 2006;87:60-66.
5. Csardi G, Nepusz T. The igraph software package for complex network research. InterJournal, Complex Systems 1695. 2006. <http://igraph.org>
6. R Core Team (2018). R : A language and environment for statistical computing. R Foundation for Statistical Computing, Vienna, Austria. URL <https://www.R-project.org/>.
7. Wold S, ed. PLS for multivariate linear modelling*.* Weinheim, Germany: Verlag Chemie: 1995.
8. Wold S, Sjostrom M, Eriksson L. PLS-regression: a basic tool of chemometrics. *Chemometrics and Intelligent Laboratory Systems* 2001; 58(2):109-130.
9. Mevik BH, Wehrens R, Liland KH (2018). Pls: Partial Least Squares and Principle Component Regression. R package version 2.7-0. <https://CRAN.R-project.org/package-pls>.
10. Efron B, Gong G. A leisurely look at the bootstrap, jack-knife and cross-validation. The *American* *Statistician.* 1983;37:36-48.
